# Supplementary material for: Skeletal muscle transcriptome in healthy aging
Source: Nat Commun. 2021 Apr 1;12:2014. doi: 10.1038/s41467-021-22168-2 (PMC8016876; doi:10.1038/s41467-021-22168-2)
Supplement: Supplementary file 1 — Supplementary Information [file 41467_2021_22168_MOESM1_ESM.pdf]

Skeletal Muscle Transcriptome in Healthy Aging

Robert A. Tumasian III, Abhinav Harish, Gautam Kundu, Jen-Hao Yang, Ceereena Ubaida-Mohien, Marta Gonzalez-Freire, Mary Kaileh, Linda M. Zukley, Chee W. Chia, Alexey Lyashkov, William H. Wood III, Yulan Piao, Christopher Coletta, Jun Ding, Myriam Gorospe, Ranjan Sen, Supriyo De & Luigi Ferrucci*

National Institute on Aging – Intramural Research Program
National Institutes of Health
Baltimore, MD 21224, USA

*Correspondence: FerrucciLu@grc.nia.nih.gov

Keywords: RNA variants; alternative splicing; transcriptomics; proteomics; skeletal muscle; aging; senescence; myogenesis

Supplementary References

111. Tanaga, Kousei, et al. "LRP1B attenuates the migration of smooth muscle cells by reducing membrane localization of urokinase and PDGF receptors." *Arteriosclerosis, thrombosis, and vascular biology* **24.8** (2004): 1422-1428.

112. Poduslo, S.E., R. Huang, and A. Spiro, *A Genome Screen of Successful Aging Without Cognitive Decline Identifies LRP1B by Haplotype Analysis.* American Journal of Medical Genetics Part B-Neuropsychiatric Genetics, 2010. **153b**(1): p. 114-119.

113. Gamazon, Eric R., et al. "Comprehensive genetic analysis of cytarabine sensitivity in a cell-based model identifies polymorphisms associated with outcome in AML patients." *Blood* (2013): blood-2012.

114. Prodi, Dionigio Antonio, et al. "EDA2R is associated with androgenetic alopecia." *Journal of investigative dermatology* **128.9** (2008): 2268-2270.

115. Nowick, Katja, et al. "Gain, loss and divergence in primate zinc-finger genes: a rich resource for evolution of gene regulatory differences between species." *PLoS One* **6.6** (2011): e21553.

116. Moni, Mohammad Ali, and Pietro Lio'. "Genetic profiling and comorbidities of Zika infection." *The Journal of infectious diseases* **216.6** (2017): 703-712.

117. Pehmøller, Christian, et al. "Genetic disruption of AMPK signaling abolishes both contraction-and insulin-stimulated TBC1D1 phosphorylation and 14-3-3 binding in mouse skeletal muscle." *American Journal of Physiology-Endocrinology and Metabolism* **297.3** (2009): E665-E675.

118. St-Amand, Jonny, et al. "Effects of mild-exercise training cessation in human skeletal muscle." *European journal of applied physiology* **112.3** (2012): 853-869.

119. Wainer-Katsir, Kerem, James Y. Zou, and Michal Linial. "Extended fertility and longevity: the genetic and epigenetic link." *Fertility and sterility* **103.5** (2015): 1117-1124.

120. Das, Swapan Kumar, et al. "Calsquestrin 1 (CASQ1) gene polymorphisms under chromosome 1q21 linkage peak are associated with type 2 diabetes in Northern European Caucasians." *Diabetes* **53.12** (2004): 3300-3306.

121. Rossi, Daniela, et al. "A Mutation in the CASQ1 Gene Causes a Vacuolar Myopathy with Accumulation of Sarcoplasmic Reticulum Protein Aggregates." *Human mutation* **35.10** (2014): 1163-1170.

a

Characteristic	All Subjects (n=53)	Age ≤ median* (n=28)	Age > median* (n=25)	p†
Male, n (%)	33 (62.3)	17 (60.7)	16 (64.0)	1
Black, n (%)	12 (22.6)	10 (35.7)	2 (8.0)	0.022
Education (highest completed), n (%)				0.457
>12th grade	30 (56.6)	14 (50.0)	16 (64.0)	
>16th grade	13 (24.5)	7 (25.0)	6 (24.0)	
BMI, mean (sd)	25.8 (2.8)	26.1 (2.6)	25.5 (3.0)	0.414
Waist circumference‡ , mean (sd)	88.3 (9.6)	85.6 (7.8)	91.3 (10.7)	0.039
Systolic blood pressure, mean (sd)	119 (13)	116 (10)	122 (14)	0.066
RMR‡ , mean (sd)	1372 (233)	1432 (187)	1304 (265)	0.066
VO2 max‡, mean (sd)	29.0 (6.2)	32.0 (5.9)	25.6 (4.6)	<0.001
Fasting glucose, mean (sd)	89.2 (8.6)	86.8 (6.0)	91.8 (10.3)	0.037
400m walk time (s), mean (sd)	250 (30)	241 (21)	261 (35)	0.015
Knee strength◊ (Nm), mean (sd)	191 (63)	213 (58)	167 (61)	0.007

* Median age in study sample is 52 yr, in which four participants are 52 yr. Age range in study is 22-83 yr.
† Fisher's exact χ^2 test or t-test
‡ $n_{waist\ circumference} = 51$, $n_{RMR} = 47$, $n_{VO2\ max} = 42$
◊ Left quadriceps 30 deg/s concentric peak torque. Right quadriceps 30 deg/s concentric peak torque used for one participant (31 yr). One participant (28 yr) with no quadriceps data available was removed.

b

	Protein-coding	Non-coding and others	Total
Mean	15,291.4	9,162.08	24,453.47
Standard Deviation	1,758.79	2,990.68	4,610.29
Minimum	11,517	4,686	16,203
Maximum	18,966	17,339	36,119
Total (in all samples)	19,970	29,159	49,129
Percentage	98.24	77.87	85.04

c

	Protein-coding	Non-coding and others	Total
Mean	27,907.59	38,075.4	66,632.98
Standard Deviation	4,527.43	10,400.17	14,728.25
Minimum	20,954	21,768	42,928
Maximum	40,371	69,753	110,124
Total (in all samples)	68,349	97,203	165,552
Percentage	83.61	84.81	84.31

Supplementary Fig. 1. Participant characteristics and RNA (ENSG) / isoform (ENST) detection. a Participants (n=53) were divided into two age groups (22-52 yr, n=28; 53-82 yr, n=25). Mean values and standard deviations are provided for all continuous variables. Group-wise quantities and percent frequencies are provided for each categorical characteristic. A Fisher's exact test (for categorical variables) or t-test (for continuous variables) (all two-sided, unadjusted) was used to determine if there was a significant ($p<0.05$) difference in patient characteristics between the two age groups. BMI, RMR and VO2 max denote body mass index, resting metabolic rate and maximal oxygen uptake, respectively. **b** Average number of protein-coding and non-coding RNAs detected per sample, including the percentage of RNAs identified in the Ensembl hg19 v82 (September 2015) database. **c** Average number of protein-coding and non-coding isoforms detected per sample, including the percentage of isoforms identified in the Ensembl hg19 v82 (September 2015) database.

a

	Rank	RNA Ensembl ID (common name)	P-value	Beta	Associated Functions & Characteristics
Protein-Coding	1	*ENSG00000147883* ***(CDKN2B)***	7.14E-11	0.0217	Aging[9], p15 tumor suppressor[22], aneurysm formation[63]
	2	*ENSG00000168143* ***(FAM83B)***	1.03E-10	0.0231	Hypoxia response pathway[14], aging, skeletal muscle weakness[18]
	3	*ENSG00000108231* ***(LGI1)***	4.59E-09	0.0199	Aging, skeletal muscle weakness[18], limbic encephalitis[84], epilepsy[85]
	4	*ENSG00000089101* ***(CFAP61)***	1.2E-08	0.0221	High expression in old skeletal muscle, aging, skeletal muscle weakness[18]
	5	*ENSG00000005020* ***(SKAP2)***	3.08E-08	0.0153	Sarcomere function and regulation, higher expression in older subjects[16]
	7	*ENSG00000235162* ***(C12orf75)***	9.38E-08	0.0269	Energy metabolism, insulin signaling[15], aging, skeletal muscle weakness[18]
	8	*ENSG00000150938* ***(CRIM1)***	2.28E-07	0.0142	Skeletal muscle aging[16], smooth muscle contractility[17], CNS development[86]
	9	*ENSG00000083720* ***(OXCT1)***	2.28E-07	0.0204	Related to energy production from ketone bodies[25]
	10	*ENSG00000185551* ***(NR2F2)***	2.37E-07	0.0110	Myogenesis, skeletal muscle development[12], aging[23], muscular dystrophy[87]
	11	*ENSG00000168702* ***(LRP1B)***	3.48E-07	0.0229	Attenuates the migration of smooth muscle cells[111], aging without cognitive decline[112]
	12	*ENSG00000132464* ***(ENAM)***	4.09E-07	0.0107	Tooth enamel formation[95]
	13	*ENSG00000145012* ***(LPP)***	6.93E-07	0.0091	Smooth muscle differentiation[13], cell migration and proliferation[88]
	15	*ENSG00000196482* ***(ESRRG)***	8.01E-07	0.0133	Type I muscle fiber development, estrogen signaling[34], obesity susceptibility[35]
	16	*ENSG00000131080* ***(EDA2R)***	1.13E-06	0.0261	Involved in p53 mediated apoptosis[113], male hair loss[114]
	17	*ENSG00000185760* ***(KCNQ5)***	1.56E-06	0.0114	Potassium channel subunit[93]
	18	*ENSG00000138439* ***(FAM117B)***	1.84E-06	0.0120	Sarcoidosis susceptibility[96], candidate for early onset myocardial infarction[97]
	20	*ENSG00000223547* ***(ZNF844)***	2.06E-06	0.0131	Pronounced expression in testis[115], transcription regulator[116]
Non-Coding	6	*ENSG00000264151*	7.76E-08	0.0277	lincRNA
	14	*ENSG00000263405*	7E-07	0.0257	Proliferation-associated pseudogene
	19	*ENSG00000230069*	1.94E-06	0.0166	Leucine-rich repeat pseudogene

b

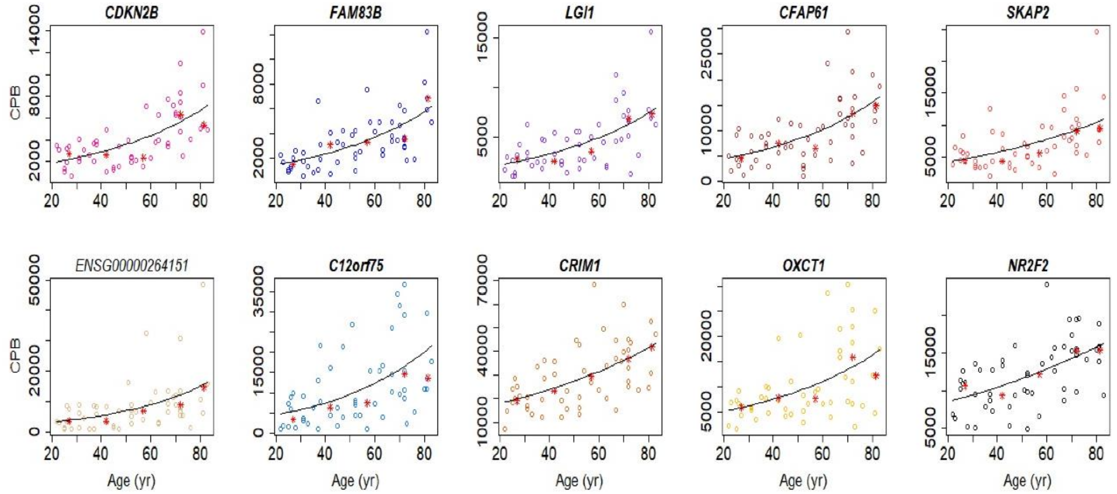

Supplementary Fig. 2. Top 20 significant (p<0.01) RNAs (ENSGs) identified by negative binomial regression with positive beta values for age. a Negative binomial model p-values (two-sided Wald tests, unadjusted) and beta values, and RNA -gene function from literature review. **b** Negative binomial plots representing CPB values with age for the top ten RNAs ranked by lowest p-value (two-sided Wald tests, unadjusted). Red asterisks represent median CPB values within the five age groups.

a

	Rank	RNA Ensembl ID (common name)	P-value	Beta	Associated Functions & Characteristics
Protein-Coding	1	*ENSG00000134333* ***(LDHA)***	7.14E-11	-0.0166	Porcine energy regulation, glycogen metabolism, and glycolysis of skeletal muscle[98]
	3	*ENSG00000140416* ***(TPM1)***	4.59E-09	-0.0169	Modulates actin-myosin interactions[32]
	4	*ENSG00000180209* ***(MYLPF)***	1.2E-08	-0.0154	Fast and slow skeletal muscle development[29], lower in old caprine skeletal muscle[108]
	6	*ENSG00000170290* ***(SLN)***	7.76E-08	-0.0117	Inhibits rat sarcoplasmic Ca^{2+}-ATPases[102], thermogenesis[103], muscle performance[104]
	7	*ENSG00000111669* ***(TPI1)***	9.38E-08	-0.0133	Glycolysis enzyme, energy generation for muscle cells in chickens[101]
	8	*ENSG00000197872* ***(FAM49A)***	2.28E-07	-0.0106	Increased proteomic expression with age in mice[99], dementia[100]
	9	*ENSG00000145949* ***(MYLK4)***	2.28E-07	-0.0175	Aging, weakness[18], contraction[19], motility[20], TGF-beta pathway[21], circadian rhythms[106]
	10	*ENSG00000130595* ***(TNNT3)***	2.37E-07	-0.0122	Skeletal muscle contractibility[30], porcine muscle development, tropomyosin binding[31]
	13	*ENSG00000065882* ***(TBC1D1)***	6.93E-07	-0.0102	Inhibits insulin-stimulated GLUT4 translocation, muscle contraction[117]
	14	*ENSG00000168530* ***(MYL1)***	7E-07	-0.0092	Expressed in fast-type muscle[14]
	16	*ENSG00000103145* ***(HCFC1R1)***	1.13E-06	-0.0152	Cellular movement[118], metabolic signaling and aging[119]
	17	*ENSG00000166343* ***(MSS51)***	1.56E-06	-0.0102	Skeletal muscle-specific modulator of cellular metabolism[110]
	19	*ENSG00000183785* ***(TUBA8)***	1.94E-06	-0.0131	Intracellular transport of macromolecules and cytoskeletal support[107]
	20	*ENSG0000014331* ***(CASQ1)***	2.06E-06	-0.0125	Type 2 diabetes[120], accumulation of sarcoplasmic reticulum protein aggregates[121]
Non-Coding	2	*ENSG00000234281*	1.03E-10	-0.0186	LANCL1 antisense RNA
	5	*ENSG00000257542*	3.08E-08	-0.0139	Olfactory receptor pseudogene
	11	*ENSG00000248923*	3.48E-07	-0.0253	MT-ND5 pseudogene
	12	*ENSG00000256364*	4.09E-07	-0.0109	MLEC antisense RNA
	15	*ENSG00000270136*	8.01E-07	-0.0097	MINOS1 readthrough gene (nonsense-mediated decay)
	18	*ENSG00000259032*	1.84E-06	-0.0161	Endosulfine alpha pseudogene

b

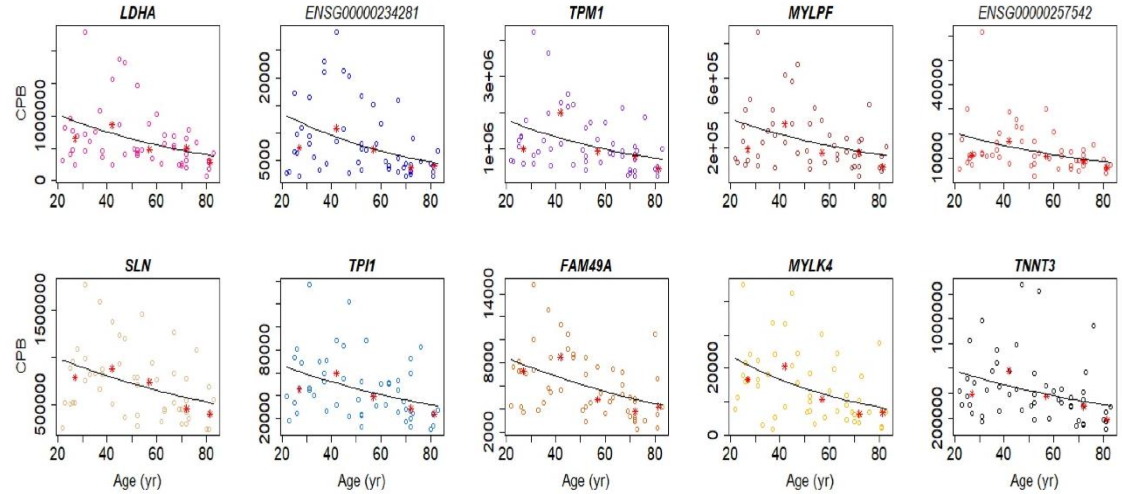

Supplementary Fig. 3. Top 20 significant (p<0.01) RNAs (ENSGs) identified by negative binomial regression with negative beta values for age. a Negative binomial model p-values (two-sided Wald tests, unadjusted) and beta values, and RNA -gene function from literature review. **b** Negative binomial plots representing CPB values with age for the top ten RNAs ranked by lowest p-value (two-sided Wald tests, unadjusted). Red asterisks represent median CPB values within the five age groups.

a

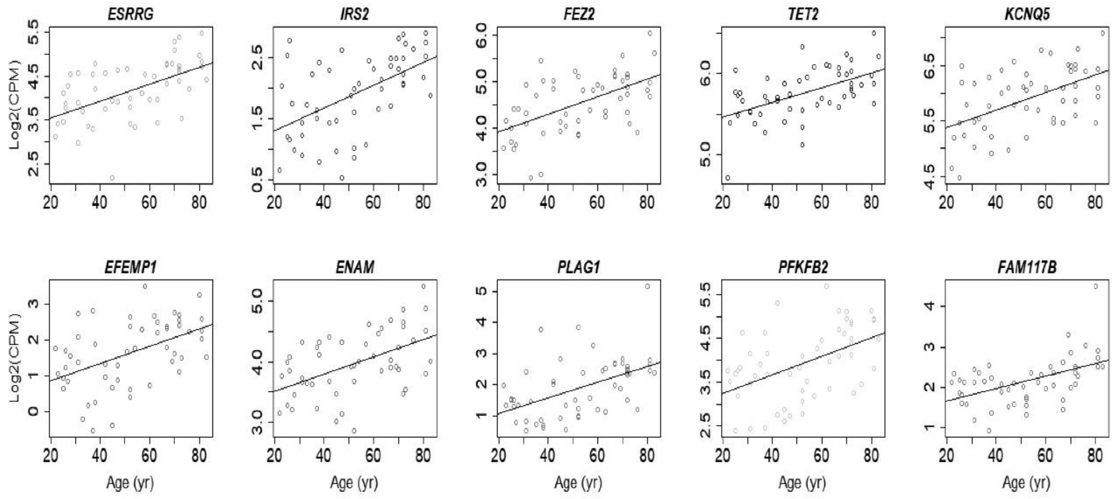

b

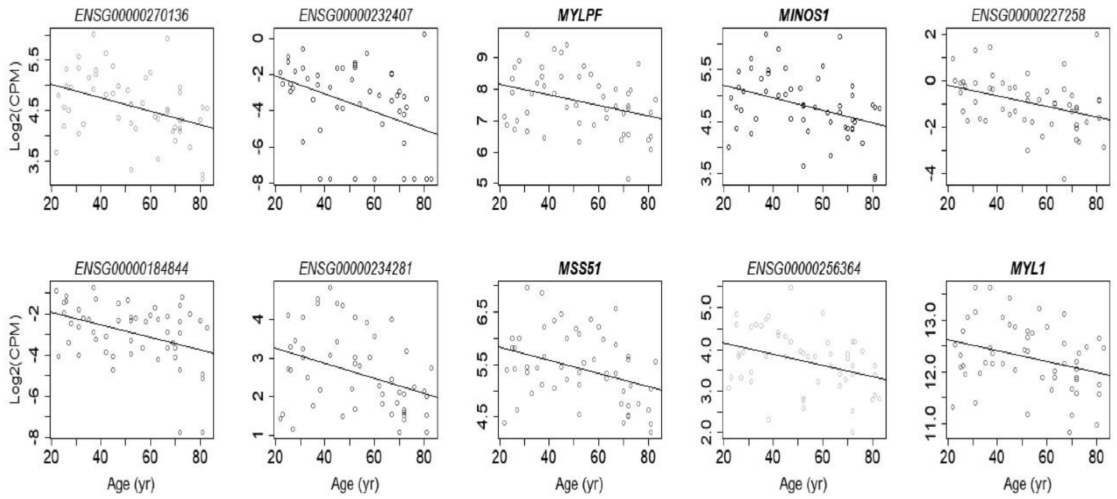

Supplementary Fig. 4. Linear model plots for RNAs (ENSGs) ranked 11-20 by lowest p-value. RNAs that failed to have at least ten samples with CPM values above -3 were removed. **a** Expression patterns of RNAs obtained from linear regression analysis (two-sided Wald tests, unadjusted) with positive beta values for age. **b** Expression patterns of RNAs obtained from linear regression analysis (two-sided Wald tests, unadjusted) with negative beta values for age.

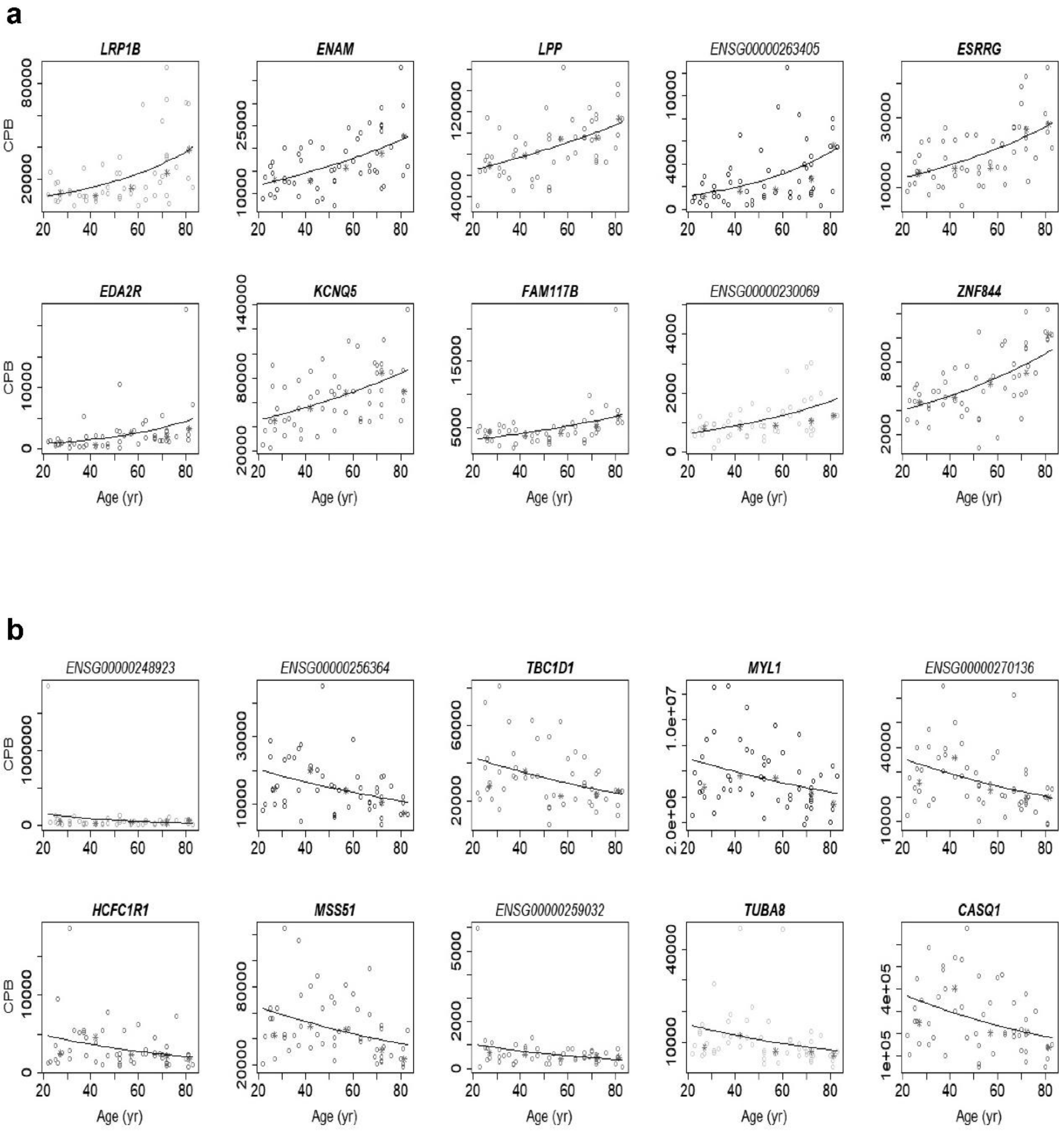

Supplementary Fig. 5. Negative binomial model plots for RNAs (ENSGs) ranked 11-20 by lowest p-value. a Expression patterns of RNAs obtained from negative binomial regression (two-sided Wald tests, unadjusted) with positive beta values for age. **b** Expression patterns of RNAs obtained by negative binomial regression (two-sided Wald tests, unadjusted) with negative beta values for age.

RNA Ensembl ID (common name)	Proteomic Data Available (Y/N)	Directionality of Protein Abundance with Age
ENSG00000147883 ***(CDKN2B)***	N	
ENSG00000168143 ***(FAM83B)***	N	
ENSG00000235162 ***(C12orf75)***	N	
ENSG00000108231 ***(LGI1)***	N	
ENSG00000005020 ***(SKAP2)***	N	
ENSG00000150938 ***(CRIM1)***	N	
ENSG00000089101 ***(CFAP61)***	N	
ENSG00000185551 ***(NR2F2)***	N	
ENSG00000145012 ***(LPP)***	Y	UP
ENSG00000196482 ***(ESRRG)***	N	
ENSG00000185950 ***(IRS2)***	N	
ENSG00000171055 ***(FEZ2)***	N	
ENSG00000168769 ***(TET2)***	N	
ENSG00000185760 ***(KCNQ5)***	N	
ENSG00000115380 ***(EFEMP1)***	Y	UP
ENSG00000132464 ***(ENAM)***	N	
ENSG00000181690 ***(PLAG1)***	N	
ENSG00000123836 ***(PFKFB2)***	Y	UP
ENSG00000138439 ***(FAM117B)***	N	
ENSG00000083720 **(OXCT1)**	Y	UP
ENSG00000168702 **(LRP1B)**	N	
ENSG00000131080 **(EDA2R)**	N	
ENSG00000223547 **(ZNF844)**	N	
ENSG00000134333 **(LDHA)**	Y	DOWN
ENSG00000197872 **(FAM49A)**	Y	DOWN
ENSG00000140416 **(TPM1)**	Y	DOWN
ENSG00000111669 **(TPI1)**	Y	DOWN
ENSG00000170290 **(SLN)**	N	
ENSG00000130595 **(TNNT3)**	Y	DOWN
ENSG00000139656 **(SMIM2)**	N	
ENSG00000145949 **(MYLK4)**	N	
ENSG00000183785 **(TUBA8)**	Y	DOWN
ENSG00000180209 **(MYLPF)**	Y	DOWN
ENSG00000173436 **(MINOS1)**	N	
ENSG00000166343 **(MSS51)**	N	
ENSG00000168530 **(MYL1)**	Y	DOWN
ENSG00000065882 **(TBC1D1)**	Y	DOWN
ENSG00000103145 **(HCFC1R1)**	N	
ENSG00000014331 **(CASQ1)**	Y	DOWN

Supplementary Fig. 6. Distinct protein-coding transcripts among our four top 20 lists. RNA Ensembl IDs (ENSGs), common names, proteomic data availability, and directionality of protein abundance with age are shown. Of the 39 distinct protein-coding transcripts obtained by our models, 14 had proteomic data available from the same muscle, of which four had higher proteomic abundance and ten had lower proteomic abundance with older age.

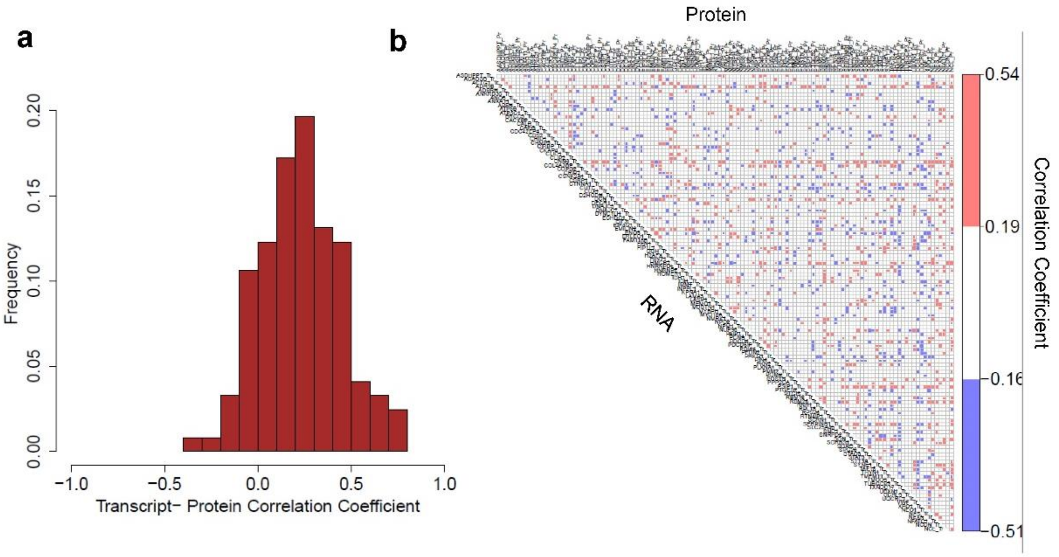

c

	Gene names	Correlation coefficient	P-value
Muscle Protein	MYOZ2	0.753661	2.67E-10
	PDLIM1	0.68	5.6E-08
	SNTB2	0.623781	1.3E-06
	MYLK3	0.469241	0.000587
	TNNT3	0.440203	0.001379
	CAV1	0.390778	0.005018
	LTBP2	0.390705	0.005027
	TPM1	0.388331	0.005324
	TNNI2	0.377104	0.006943
	MYLPF	0.349661	0.012808
	PPP1R12A	0.330236	0.019171
	MYL1	0.318926	0.023982
	CEP170	0.254192	0.074858
	TNNC2	0.251765	0.077774
	CALD1	0.248361	0.082013
	MYH2	0.248223	0.082189
	MYH11	0.224111	0.117677
	MTSS1	0.204897	0.153468
	TUBA8	0.191745	0.182215
	SORBS2	0.154866	0.282884
	MYH7B	-0.0551	0.703885

Supplementary Fig. 7. Protein vs. mRNA (ENSG) correlation analysis. a Protein vs. mRNA correlation distribution of 122 age-associated RNAs. **b** Heatmap of 122 randomly selected proteins and mRNAs, protein on y-axis and RNA on x-axis. Color-coded correlation coefficient is shown. **c** Table shows functional annotation of 16 proteins from 122 age-associated RNAs annotated as muscle function, of which 15 were positively correlated with their mRNA levels (all p-values obtained from two-sided Wald tests, unadjusted).

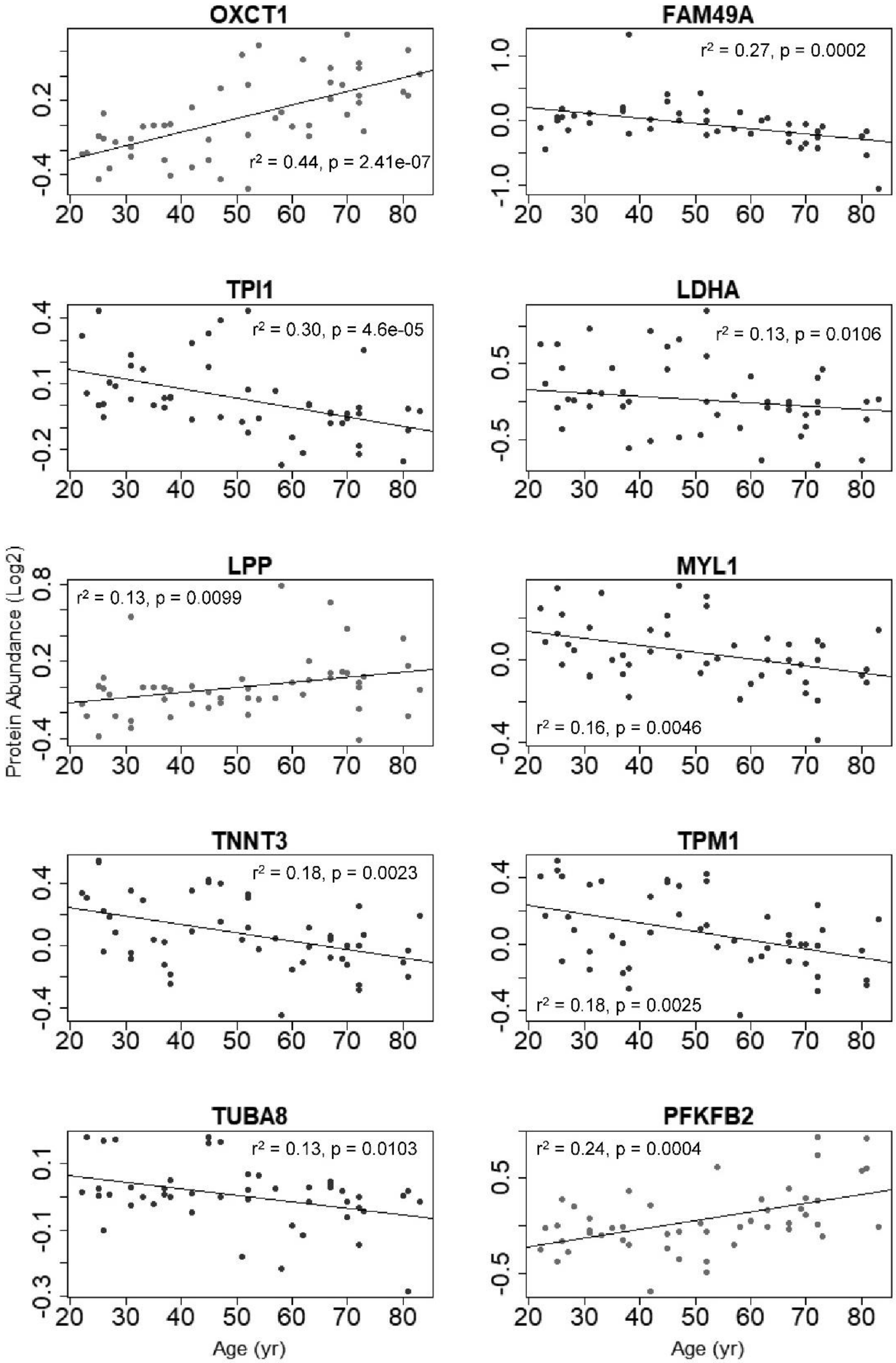

Supplementary Fig. 8. Proteomic analysis. Among the protein-coding RNAs (ENSGs) in the four top 20 lists, statistically significant (all p<0.05 from two-sided Wald tests, unadjusted)) downstream correlations between relative protein abundance and age were identified for ten mRNAs: *OXCT1*, *FAM49A*, *TPI1*, *LDHA*, *LPP*, *MYL1*, *TNNT3*, *TPM1*, *TUBA8*, and *PFKFB2* mRNAs. Red and blue points indicate mRNAs with positive and negative correlations, respectively.

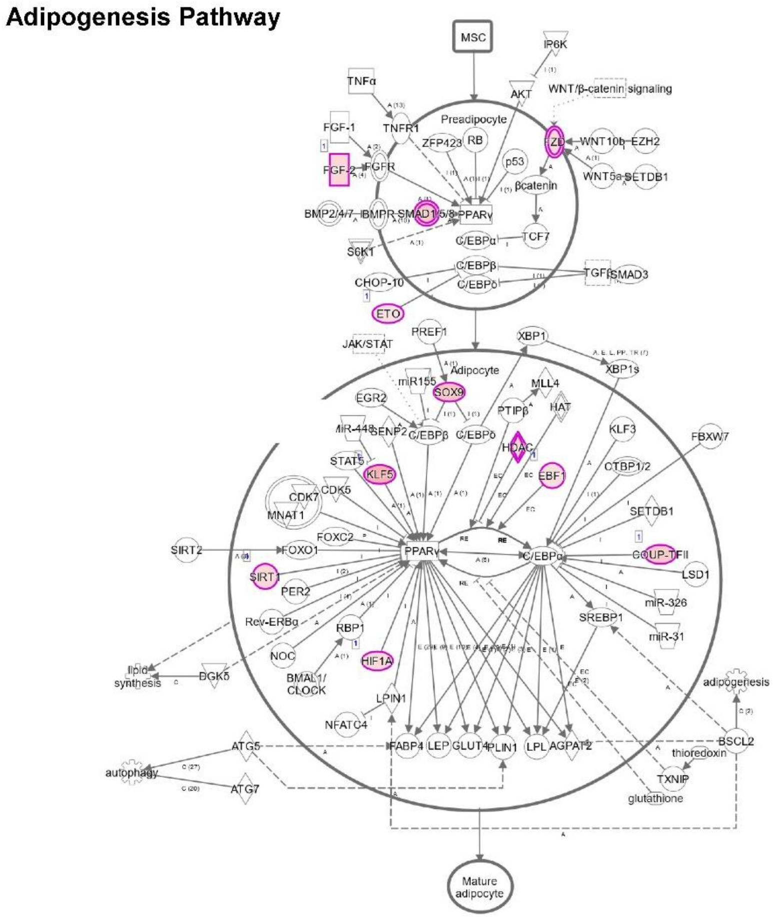

Supplementary Fig. 9. Pathways associated with the significant (p<0.01) RNAs (ENSGs) obtained by linear and negative binomial regression. IPA identified statistically significant (p<0.05) biological pathways associated with 478 significant mRNAs obtained by both the linear and negative binomial models that were present in the IPA database. Of our top RNAs, 12 mRNAs [*EBF1*, *FGF2*, *FZD5* (component of the FZD complex), *HIF1A*, *KLF5*, *NR2F2* (COUP-TFII), *RUNX1T1* (ETO), *SAP30* and *SAP130* (members of the HDAC complex, histone deacetylases), *SIRT1*, *SMAD9* (component of the SMAD complex), and *SOX9*] were matched to the 132 total genes involved in the KEGG pathway for adipogenesis (highlighted in pink). Nodes indicate protein functions of the genes annotated to the adipogenesis pathway, including G-protein coupled receptors (vertical rectangle), complexes (nested circle), transcription regulators (horizontal ellipse), transmembrane receptors (vertical ellipse), enzymes (vertical diamond), and others (circle).

a

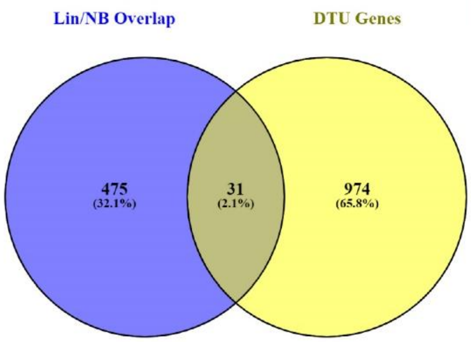

b

Gene	Number of Significant Variants (p < 0.05)		P-value	Beta
CFAP61	1	ENST00000377309	0.0050	0.0282
ESRRG	2	ENST00000366938	0.0071	0.0377
		ENST00000586199	0.0045	0.0937
TET2	1	ENST00000265149	0.0021	0.1103
PLAG1	1	ENST00000423799	0.0057	-0.3787
NEDD4L	1	ENST00000592097	0.0066	-0.0708
HERPUD2	1	ENST00000413517	0.0098	0.2915
SPOCK1	1	ENST00000503916	0.0072	-0.2464
TNRC6C	1	ENST00000585438	0.0036	0.0984
SAP130	1	ENST00000259235	0.0009	0.1709
KCNMA1	1	ENST00000604624	0.0089	-0.0824
RUNX1T1	1	ENST00000520047	0.0017	-0.0828
RAB30	1	ENST00000534103	0.0056	-0.0711
PEX13	1	ENST00000444100	0.0055	-0.0891
H2BC9	1	ENST00000356350	0.0044	1.0136
TPM1	3	ENST00000334895	0.0032	0.0029
		ENST00000558314	0.0036	0.0009
		ENST00000560975	0.0053	0.0008
ENSG00000249599	1	ENST00000510795	0.0008	1.1471
PIK3IP1	1	ENST00000493034	0.0017	-0.4265
ZNF614	1	ENST00000597952	0.0074	-0.0821
ZNF562	1	ENST00000293648	0.0062	-0.3208
TMEM158	1	ENST00000503771	0.0071	0.7113
GNPNAT1	1	ENST00000554421	0.0054	-0.1790
RNF217	1	ENST00000359704	0.0093	0.0413
EIF1AD	1	ENST00000527051	0.0068	-0.0401
CCDC34P1	1	ENST00000507441	0.0087	0.9421
GAB1	1	ENST00000509992	0.0038	-0.0261
ERLIN2	1	ENST00000519872	0.0006	-0.1546
GLIPR1L2	1	ENST00000378692	0.0058	0.0844
SEPP1	1	ENST00000508937	0.0075	-0.0392
PDXDC2P	1	ENST00000533264	0.0084	0.3586
ENSG00000267326	1	ENST00000589274	0.0055	0.5396
ENSG00000270031	1	ENST00000602843	0.0042	0.9964

Supplementary Fig. 10. Significant (p<0.01) changes in differential transcript usage of splice variants (ENSTs) with age. a Overlap of RNAs with significant (all p<0.01 from two-sided Wald test, unadjusted) changes in absolute expression levels with age obtained by linear and negative binomial regression (blue), and RNAs with at least one significantly (all p<0.01 from two-sided Wald test, unadjusted) differentially expressed transcript with age on the percentage scale (yellow), where overlap is shown in brown. **b** A total of 31 variants of protein-coding RNAs had statistically significant (all p<0.01) changes in differential transcript usage with age. Ensembl v82 (September 2015) variant IDs and linear model p-values (from two-sided Wald tests, unadjusted) and beta values are shown.

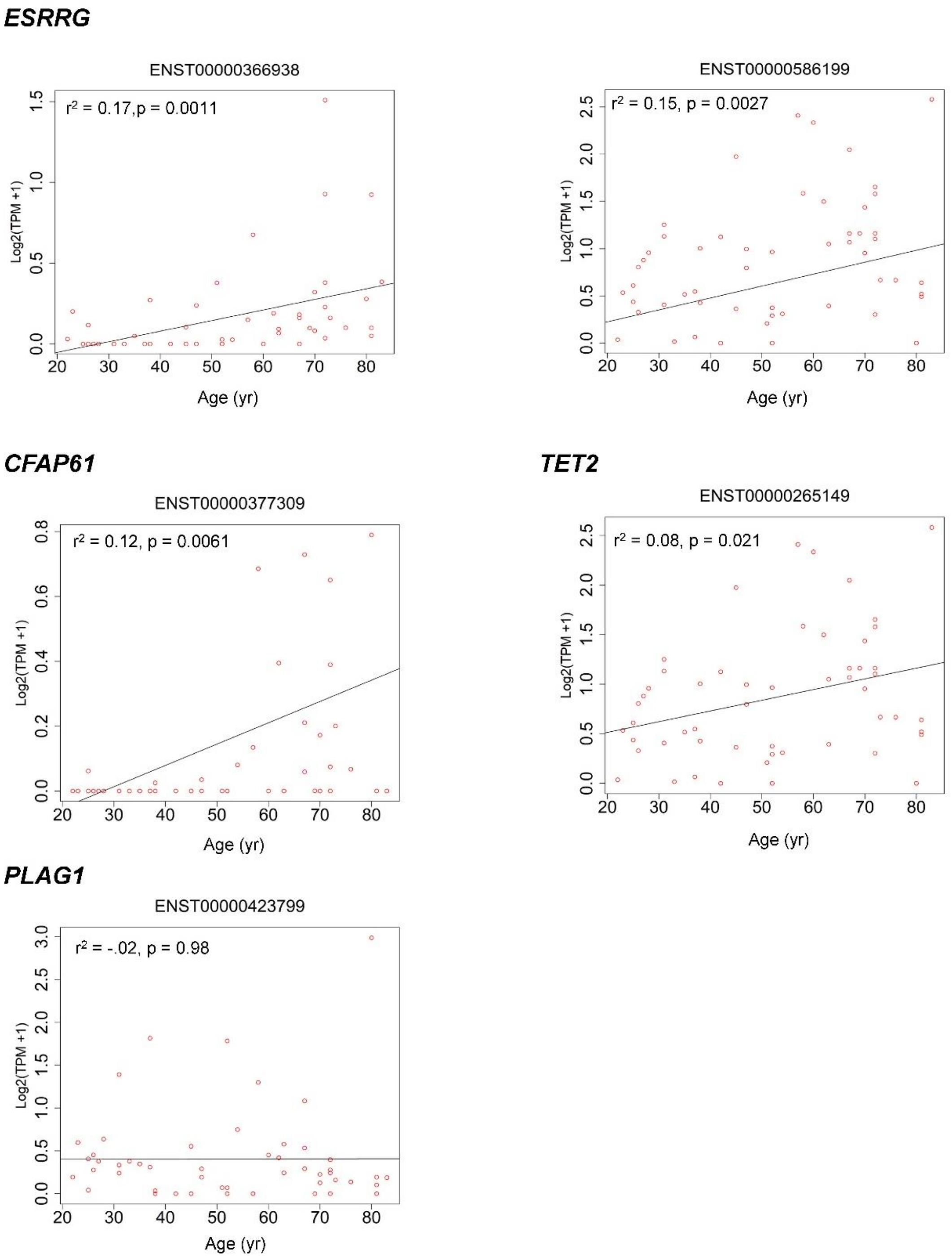

Supplementary Fig. 11. Absolute expression level changes with age for five of the 31 splice variants (ENSTs) with significantly altered differential transcript usage with age on the percentage scale. Both variants of *ESRRG* mRNA and one variant of *CFAP61* mRNA with significant ($p<0.01$, two-sided Wald test, unadjusted) differential transcript usage on the percentage scale also had significant ($p<0.01$, two-sided Wald test, unadjusted) differential transcript expression on the log2(TPM+1) scale. However, one variant of *CFAP61* mRNA and one variant of *PLAG1* mRNA with significant ($p<0.01$, two-sided Wald test, unadjusted) differential transcript usage on the percentage scale did not have significant ($p<0.01$, two-sided Wald test, unadjusted) differential transcript expression on the log2(TPM+1) scale.

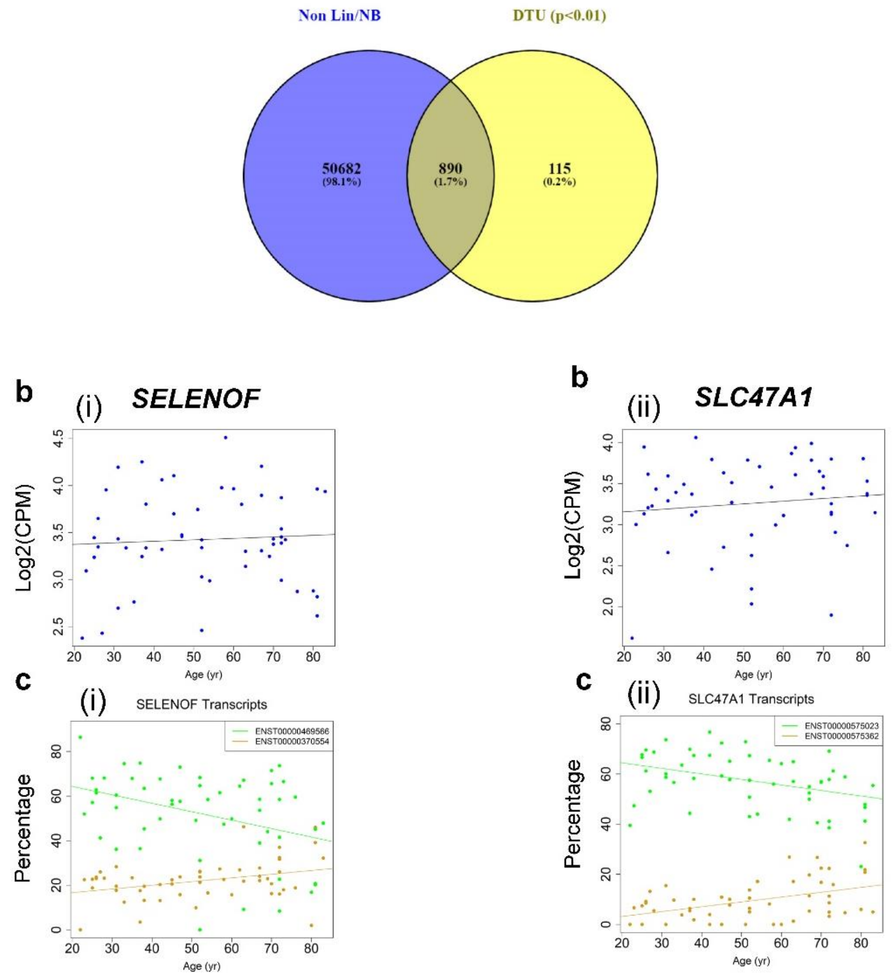

d

Gene	P-value (count model)	Beta (count model)	Number of Variants		P-value (% model)	Beta (% model)
SELENOF (ENSG00000183291)	0.6037	0.0020	2	ENST00000469566	0.0018	-0.4053
				ENST00000370554	0.0088	0.1701
SLC47A1 (ENSG00000575362)	0.3922	0.0034	2	ENST00000575023	0.0061	-0.2266
				ENST00000575362	0.0009	0.1928

Supplementary Fig. 12. Alternatively spliced variant (ENST) usage that significantly changed with age without affecting overall gene expression. **a** Overlap of the RNAs that did not significantly change with age in either the linear or NB model (blue) and had two transcripts with significantly changed usage with age (yellow), where overlap is shown in brown. **b, c** Examples chosen with high beta values, *SELENOF* and *SLC47A1*. **d** Table of p-values (two-sided Wald tests, unadjusted) and beta coefficients from selected examples.

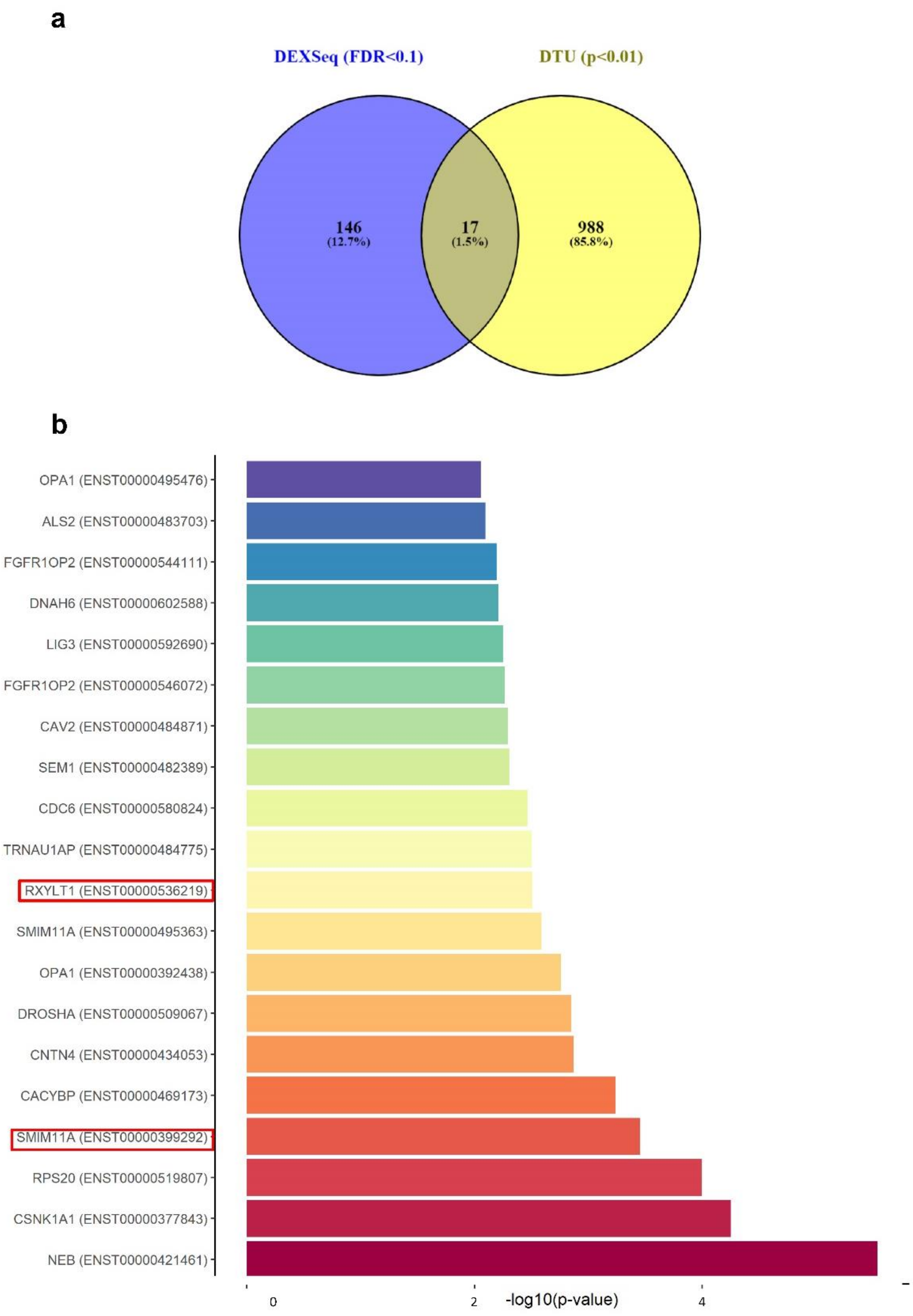

Supplementary Fig. 13. DEXseq analysis and linear regression identify mRNAs (ENSGs) with both significant exon usage and differential transcript (ENST) usage with age. a Venn diagram comparing RNAs obtained by DEXSeq (blue) and linear regression (yellow) analyses [overlap shown in brown]. DEXSeq analysis identified 146 mRNAs with significant (all FDR-adjusted p-values <0.1) changes in exon usage between the young (20-34 y, n=12) and old (80+ y, n=5) age groups. Linear regession analysis identified 988 RNAs with splice variants having significant (all p<0.01 from two-sided Wald tests, unadjusted) differential transcript usage with age on a continuous scale. Between these two analyses, there was an overlap of 17 mRNAs with significant changes in both exon usage and differential transcript usage with age. **b** Ribbon plot displaying -log10(FDR-adjusted p-values) obtained from our DEXSeq analysis for the 17 mRNAs identified by both methods, where bars are color-coded spectrally to express level of significance. Red bars indicate mRNAs with the highest statistical evidence of significant changes in exonic usage with age.

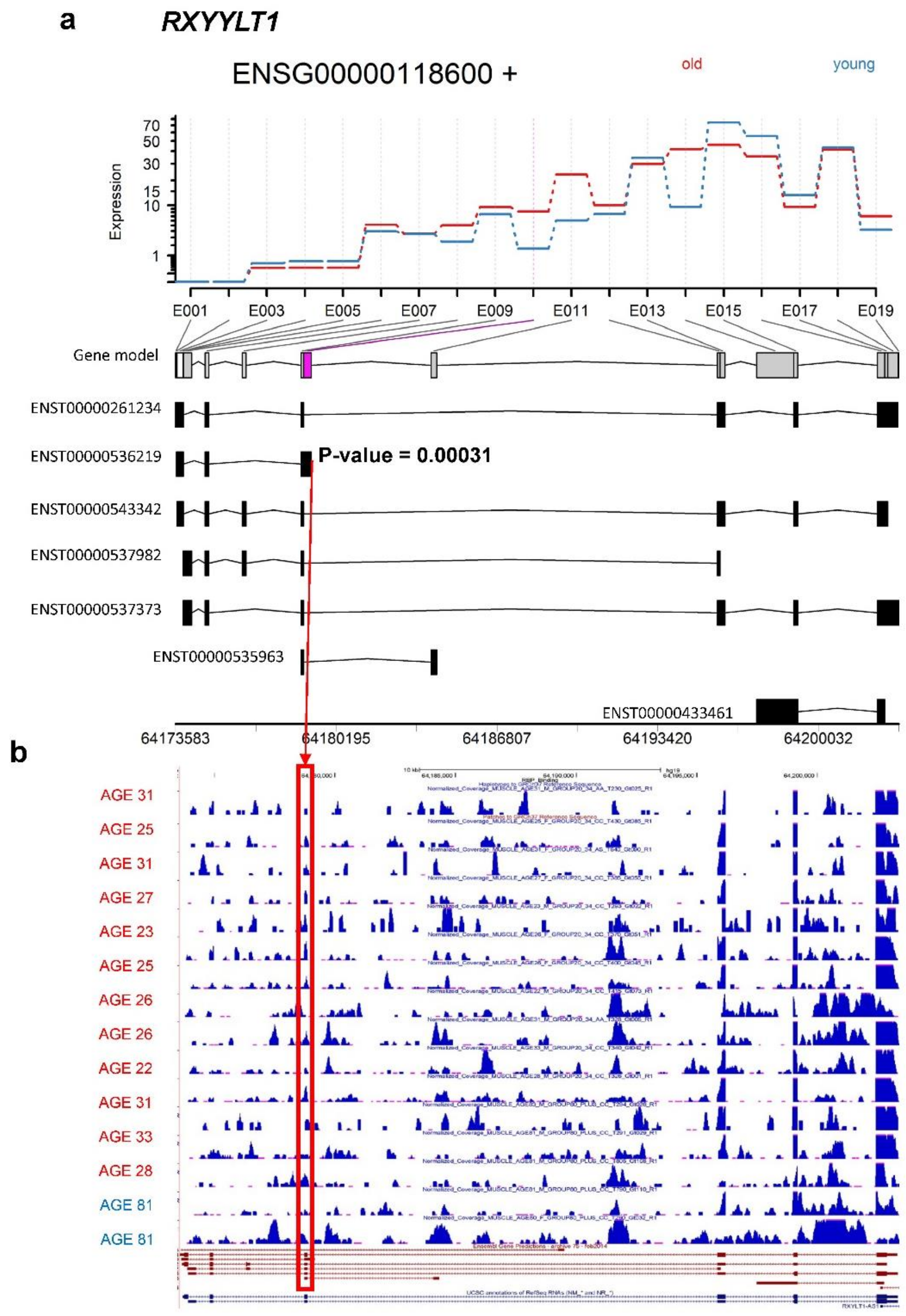

Supplementary Fig. 14. Statistically significant (p<0.01) changes in *RXYLT1* exon usage between the young (20-34 yr, n=12) and old (80+ yr, n=5) age groups. a Red and blue bars represent the average exon usage for all 19 exons of *RXYLT1* mRNA in the young and old age groups, respectively. DEXSeq analysis identified one exonic region (E010) that significantly (p<0.01) changes in absolute abundance between the age groups, labeled pink in the gene model. The average exonic usage in this region is higher in the old age group and lower in the young age group. Of the seven variants of *RXYLT1* mRNA, one has statistically significant (p<0.01 from two-sided Wald test, unadjusted) differential transcript usage with age on a continuous scale, according to our linear model. Linear model p-values are displayed for these variants. The horizontal axis below the variant models denotes the positions in the genome. **b** Ages of participants in the young (red) and old (blue) age groups are shown on the vertical axis. UCSC browser plots indicate that the significantly differentially expressed exon (location on each transcript identified by red bar) identified by our DEXSeq analysis is more expressed in the old age group than in the young age group, as indicated by the presence of larger blue peaks in older participants. UCSC browser tracks support our DEXSeq findings.

a

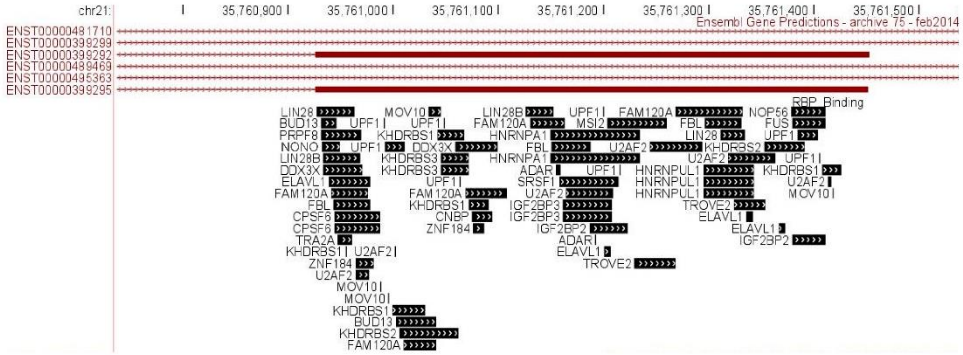

b

RNA-binding Protein	Number of Binding Sites	Associated Functions & Characteristics
**UPF1**	8	Up-frameshift suppressor, mRNA nuclear export and surveillance, nonsense-mediated mRNA decay
**U2AF2**	6	Small nuclear RNA auxiliary factor, RNA binding and splicing
**FAM120A**	5	Binding promotes *IGF2* secretion, oxidative stress-induced survival signaling, mRNA transport
**KHDRBS1**	5	Signal transduction, alternative splicing, RNA 3'-end formation, regulator of HIV gene expression
**ELAVL1**	4	mRNA degradation, highly expressed in many cancers
**MOV10**	4	RISC complex RNA helicase
**FBL**	3	Fibrillarin, pre-ribosomal RNA processing, chromatin regulation, acetylation, methyltransferase activity
**HNRNPUL1**	3	Ribonucleoprotein, mRNA splicing, RNA binding and transport
**LIN28**	2	Regulator of genes involved in developmental timing and self-renewal in embryonic stem cells
**BUD13**	2	pre-mRNA splicing
**LIN28B**	2	Negatively regulated by microRNAs that target sites in the 3' UTR, overexpressed in tumors
**DDX3X**	2	DEAD-box helicase, transcriptional regulation, mRNP assembly, pre-mRNA splicing, mRNA export
**CPSF6**	2	RNA cleavage and polyadenylation, assembly of the 3'-end processing complex
**ZNF184**	2	Zinc finger protein, gene expression, may be involved in transcriptional regulation
**KHDRBS2**	2	Signal transduction, nucleic acid binding, protein heterodimerization activity
**KHDRBS3**	2	Signal transduction, nucleic acid binding, RNA binding
**HNRNPA1**	2	Ribonucleoprotein, pre-mRNA processing, mRNA metabolism and transport, alternative splicing
**ADAR**	2	Adenosine deaminase, RNA editing
**IGF2BP3**	2	Insulin signaling, may repress *IGF2* mRNA translation during late development
**IGF2BP2**	2	Insulin signaling, regulates *IGF2* mRNA translation
**TROVE2**	2	May play roles in RNA stabilization and protection from degradation, cilia formation/maintenance
**PRPF8**	1	pre-mRNA processing and mRNA splicing
**NONO**	1	Transcriptional regulator, mRNA splicing
**TRA2A**	1	Regulation of pre-mRNA splicing
**CNBP**	1	Zinc finger protein, transcription regulator, myotonic dystrophy
**SRSF1**	1	Serine- and arginine-rich splicing factor, spinocerebellar ataxia
**MSI2**	1	Transcriptional regulator that targets genes involved in development and cell cycle regulation
**NOP56**	1	Ribonucleoprotein, pre-rRNA processing, spinocerebellar ataxia
**FUS**	1	Pre-mRNA processing, mRNA export, gene expression regulation

Supplementary Fig. 15. Proteins binding to the significantly (p<0.05) differentially expressed exon (3'UTR) of *SMIM11A* mRNA. a The horizontal axis above the red transcript tracks denotes the genomic position within chromosome 21. One splice variant of *SMIM11A* with significant differential exon usage (ENST00000399292) contains the significantly differentially expressed exon in the *SMIM11A* 3'UTR. Proteins and their binding locations are shown in black. **b** Table of proteins binding to the differentially expressed exon in the *SMIM11A* 3'UTR. Number of binding sites and functions (obtained from GeneCards) are provided.

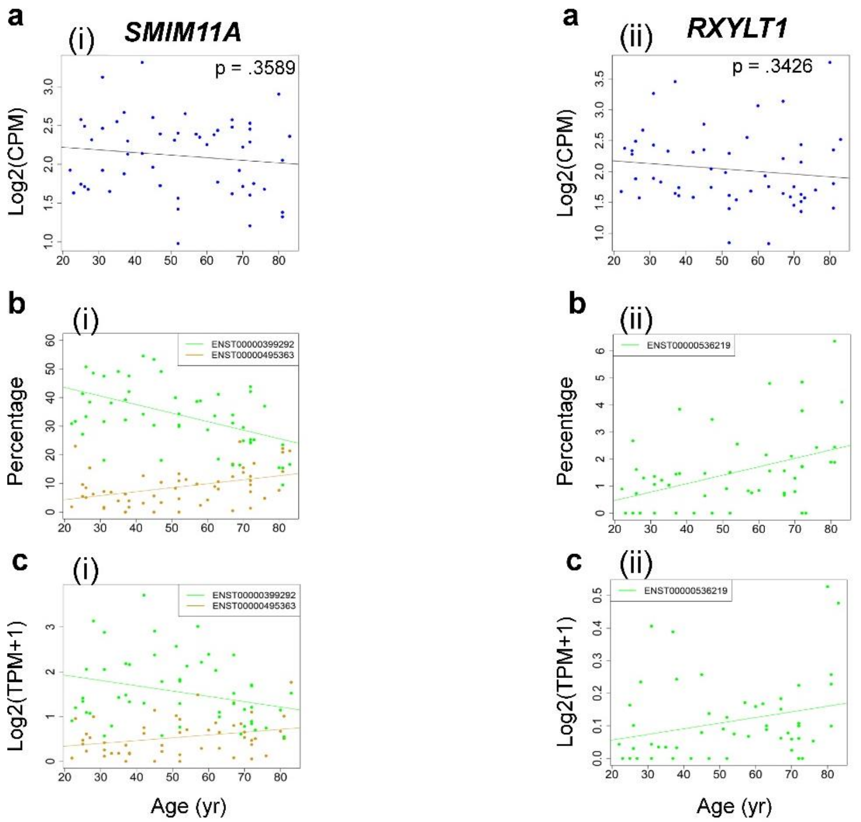

d

Gene	Number of Significant Variants (p < 0.05)		P-value (% model)	Beta (% model)	P-value (count model)	Beta (count model)
SMIM11A	2	ENST00000399292	0.0005	-0.2982	0.0359	-0.0119
		ENST00000495363	0.0024	0.1391	0.0324	0.0062
RXYLT1	1	ENST00000536219	0.0025	0.0312	0.060	0.0017

Supplementary Fig. 16. mRNAs (ENSGs) and associated splice variants (ENSTs) with significant differential transcript usage and exonic region changes with age. **a** Linear plots displaying the absolute expression patterns [log2(CPM)] of two top RNAs obtained by DEXSeq and linear regression analyses with age (p-values from two-sided Wald tests, unadjusted). **b** Linear plots displaying the differential transcript usage (on the percentage scale) of each statistically significant splice variant identified by our DEXSeq and linear regession analyses continuously with age. **c** Linear plots displaying the differential transcript expression [log2(TPM+1)] patterns of each statistically significant splice variant identified by DEXSeq and linear regression analyses continuously with age. **d** P-values (two-sided Wald tests, unadjusted) and beta values obtained from the linear percent change and TPM models are provided. Both significant variants of *SMIM11A* mRNA and the significant variant of *RXYLT1* mRNA had significant changes in differential transcript usage with age on the percentage scale (percent change model). While both variants of *SMIM11A* mRNA also displayed significant changes in differential transcript expression on the log2(TPM+1) scale, this was not the case for the significant variant of *RXYLT1* mRNA.

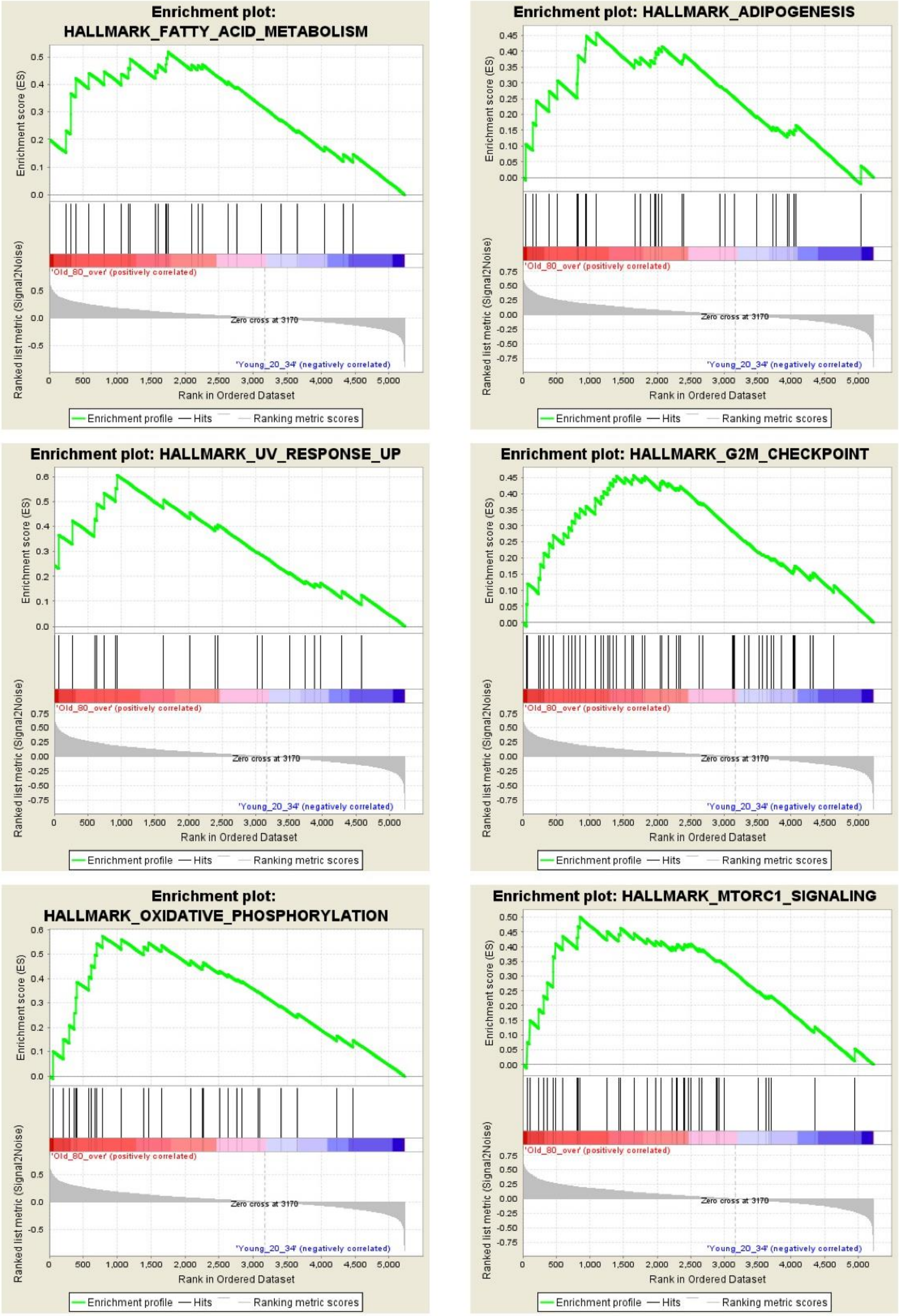

Supplementary Fig. 17. Gene Set Enrichment Analysis (GSEA) identifies significantly upregulated pathways associated with the 1,591 RNAs (ENSGs) with at least one significantly changing splice variant (ENST) with age. The fatty acid metabolism, adipogenesis, UV response, G2M ckeckpoint, oxidative phosphorylation, and MTORC1 signaling pathways were significantly (all unadjusted $p<0.05$ from two-sided Fisher Exact test) upregulated in the old (80+ yr, n=5) compared to the young (20-34 yr, n=12) participants.

a

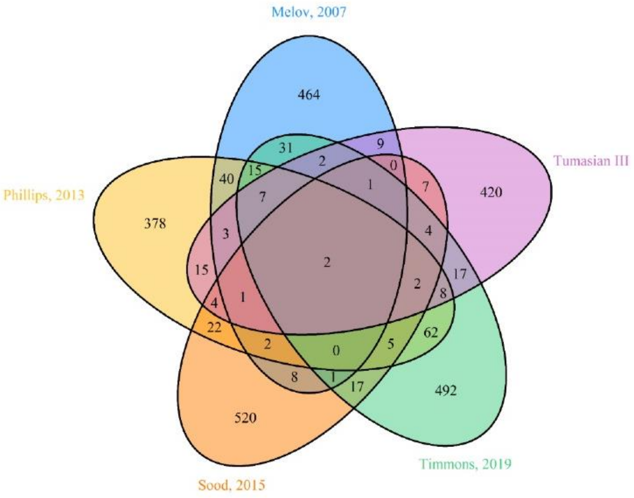

b

Paper	Melov, 2007	Phillips, 2013	Sood, 2015	Timmons, 2019	Tumasian III
**Melov, 2007**	586	70	15	59	25
**Phillips, 2013**	70	566	38	101	42
**Sood, 2015**	15	38	596	32	21
**Timmons, 2019**	59	101	32	666	43
**Tumasian III**	25	42	21	43	502

c

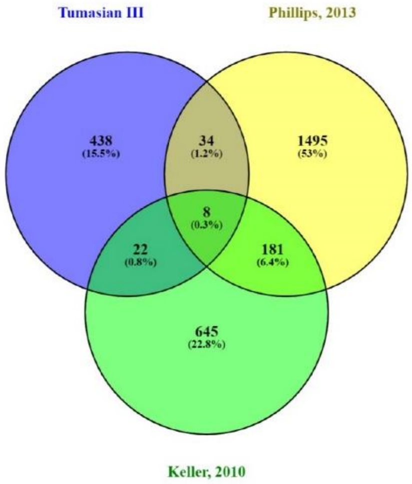

d

Paper	Keller, 2010	Phillips, 2013	Tumasian III
**Keller, 2010**	856	189	30
**Phillips, 2013**	189	1,781	42
**Tumasian III**	30	42	502

Supplementary Fig. 18. Comparison of significantly differentially expressed RNAs (ENSGs) obtained from this study and previous studies. a,**b** Overlap between the age-associated RNAs found in this study and those identified in prior large-scale RNA expression studies. A handful of transcripts overlap among these five studies (including the present study). Considering all five studies, our findings were replicated for 285 transcripts, while 82 transcripts identified as significantly changing with age in our study were replicated in at least one other study. **c**,**d** Overlap between the age-associated RNAs found in this study and those related to exercise status as identified in previous studies. Eight RNAs associated with aging in our study that were identified as impacted by exercise in both prior studies and 56 were identified as impacted by exercise by at least one of these two studies.

Supplementary primer pair information

RNA	Name of forward primer	Sequence of forward primer	Name of reverse primer	Sequence of reverse primer
CDKN2B	CDKN2B-F	GTGGGAGAAGGCAGTGATTAG	CDKN2B-R	CTCCACTTTGTCCTCAGTCTTC
FAM83B	FAM83B-F	GTGCCATACCTCCTGCTTAATA	FAM83B-R	TGTGTTGTGGTGGCTTACA
CRIM1	CRIM1-F	CAGTGGCCCAGAGGTTATTT	CRIM1-R	GTGCTCGTCTGGTAGTTCTAAG
LGI1	LGI1-F	GACACTCAGAACGCCTCATTTA	LGI1-R	GCGTACACATCCTCCATGTTAG
NR2F2	NR2F2-F	GCACGAAGGATGTGCTTCTA	NR2F2-R	CCTCACACACATAGGGAAAGAG
C12orf75	C12orf75-F	CAAGAGCTTGCTCATCAGTTTG	C12orf75-R	AAAGGGAAGGGTGACTGTTTAG
IRS2	IRS2-F	TAGGCATCAATGGGTGGTATTT	IRS2-R	CTACGGATAGAGGGCGAGTTA
SKAP2	SKAP2-F	GTTTGCAGAGGAGAGTGTAGAA	SKAP2-R	CTCAGGCAGCACAGAGTATAAC
