# Supplementary material for: Skeletal muscle transcriptome in healthy aging
Source: Nat Commun. 2021 Apr 1;12:2014. doi: 10.1038/s41467-021-22168-2 (PMC8016876; doi:10.1038/s41467-021-22168-2)
Supplement: Supplementary file 2 — Reporting Summary [file 41467_2021_22168_MOESM2_ESM.pdf]

nature research

Corresponding author(s): Dr. Luigi Ferrucci

Last updated by author(s): Jan 16, 2021

Reporting Summary

Nature Research wishes to improve the reproducibility of the work that we publish. This form provides structure for consistency and transparency in reporting. For further information on Nature Research policies, see Authors & Referees and the Editorial Policy Checklist .

Statistics

For all statistical analyses, confirm that the following items are present in the figure legend, table legend, main text, or Methods section.

n/a	Confirmed	
☐	☒	The exact sample size (*n*) for each experimental group/condition, given as a discrete number and unit of measurement
☐	☒	A statement on whether measurements were taken from distinct samples or whether the same sample was measured repeatedly
☐	☒	The statistical test(s) used AND whether they are one- or two-sided<br>*Only common tests should be described solely by name; describe more complex techniques in the Methods section.*
☐	☒	A description of all covariates tested
☐	☒	A description of any assumptions or corrections, such as tests of normality and adjustment for multiple comparisons
☐	☒	A full description of the statistical parameters including central tendency (e.g. means) or other basic estimates (e.g. regression coefficient) AND variation (e.g. standard deviation) or associated estimates of uncertainty (e.g. confidence intervals)
☐	☒	For null hypothesis testing, the test statistic (e.g. *F*, *t*, *r*) with confidence intervals, effect sizes, degrees of freedom and *P* value noted<br>*Give P values as exact values whenever suitable.*
☒	☐	For Bayesian analysis, information on the choice of priors and Markov chain Monte Carlo settings
☒	☐	For hierarchical and complex designs, identification of the appropriate level for tests and full reporting of outcomes
☒	☐	Estimates of effect sizes (e.g. Cohen's *d*, Pearson's *r*), indicating how they were calculated

Our web collection on statistics for biologists contains articles on many of the points above.

Software and code

Policy information about availability of computer code

Data collection	After RNA was extracted from skeletal muscle biopsies, cDNA libraries were prepared. Total RNA was isolated in QIAcube, QIAGEN, cDNA was synthesized using the NuGen Ovation v2 system, and Illumina libraries were generated with the TruSeq ChIP Library Preparation Kit, Set A (IP-202-1012) and Set-B (IP-202-1024). RNA was sequenced using the Illumina HiSeq 2500 sequencing system at a depth of >80 million single-end reads. After sequencing, FASTQ files were cleaned using 'cutadapt' (version 2.7). Quality was checked using the 'fastqc' program (version 0.11.8). Reads were aligned using STAR Aligner (version 2.4.0j). BAM files were processed using the 'featureCounts' function (version 1.4.6-p5) from the 'Subread' package (version 1.6.4) in RStudio (version 1.2.1335) to count reads to various genomic features, including exons, splice variants, and genes. Kallisto (version 0.44) software was used to find transcripts per million (TPM) values for splice variants.
Data analysis	All statistical analyses were completed using R (version 3.6.1) and RStudio (version 1.2.1335). The following packages were used: DEXSeq (version 1.26), edgeR (multiple versions – 3.22.5), ggplot2 (version 3.2.1), gplots (version 3.0.1.1), MASS (version 7.3-51.5), pscl (version 1.5.2), Subread (version 1.6.4), and Corrplot (0.84 library). Pathways analysis was conducted using the Ingenuity Pathway Analysis (IPA, summer 2019 release) database from QIAGEN. Enrichment analysis was completed using the Gene Set Enrichment Analysis tool (GSEA, MSigDB version 6.2) from the Broad Institute.

For manuscripts utilizing custom algorithms or software that are central to the research but not yet described in published literature, software must be made available to editors/reviewers. We strongly encourage code deposition in a community repository (e.g. GitHub). See the Nature Research guidelines for submitting code & software for further information.

Data

Policy information about availability of data

All manuscripts must include a data availability statement. This statement should provide the following information, where applicable:

- Accession codes, unique identifiers, or web links for publicly available datasets
- A list of figures that have associated raw data
- A description of any restrictions on data availability

All RNA-seq data used to generate Figures 1-3 and 6-7 and Supplementary Figures 2-5 and 9-17, is deposited in GEO (GSE164471 [https://www.ncbi.nlm.nih.gov/geo/query/acc.cgi?acc=GSE164471]), data obtained from the Ensembl hg19 v82 (September 2015) database can be located here: ftp://ftp.ensembl.org/pub/grch37/release-82. The mass spectrometry proteomics data used to generate Figure 5 and Supplementary Figures 6-8 have been deposited to the ProteomeXchange Consortium via the PRIDE partner repository with the dataset identifier PXD011967 (http://proteomecentral.proteomexchange.org/cgi/GetDataset?ID=PXD011967). Source data for this paper are provided as a Source Data file. Additional data inquiries or requests can be directed to the corresponding author [LF].

Field-specific reporting

Please select the one below that is the best fit for your research. If you are not sure, read the appropriate sections before making your selection.

- [x] Life sciences
- [ ] Behavioural & social sciences
- [ ] Ecological, evolutionary & environmental sciences

For a reference copy of the document with all sections, see nature.com/documents/nr-reporting-summary-flat.pdf

Life sciences study design

All studies must disclose on these points even when the disclosure is negative.

Sample size	This study included 53 individuals (22-83 years old) from the Genetic and Epigenetic Signatures of Translational Aging Laboratory Testing (GESTALT) study who were defined as healthy based on evaluations completed by highly experienced nurses and very strict inclusion criteria developed by the Clinical Research Unit of the National Institute on Aging. The study proposed here has never been done before, and in the absence of reliable preliminary information, this sample size should be considered as a pilot approach.
Data exclusions	All RNAs which were not expressed across any of the participants (total 568) were removed from the regression analysis (57,773 – 568 = 57,205 remaining). Of the 57,205 RNAs, there were 544 RNAs (ENSGs) removed from the negative binomial regression analysis due to an excess of zero counts and very low median expression levels that prevented us from obtaining meaningful expression trends with age. An additional 13 RNAs were removed due to very low expression levels (all median CPB<630). Only RNAs with at least ten log2(CPB) values of 125 or greater were included in the negative binomial regression results. Similarly, only RNAs with at least ten log2(CPM) values of -3 or greater were included in the linear regression results.
Replication	This study used two regression methods to obtain RNAs (ENSGs) with significant changes in expression with age.
Randomization	Age and sex were the only covariates used in this study. Participants were split into two age groups for some analyses. No randomization was required for this study.
Blinding	Since this is an observational study, there is no blinding.

Reporting for specific materials, systems and methods

We require information from authors about some types of materials, experimental systems and methods used in many studies. Here, indicate whether each material, system or method listed is relevant to your study. If you are not sure if a list item applies to your research, read the appropriate section before selecting a response.

Materials & experimental systems

n/a	Involved in the study	
x		Antibodies
x		Eukaryotic cell lines
x		Palaeontology
x		Animals and other organisms
	x	Human research participants
	x	Clinical data

Methods

n/a	Involved in the study	
x		ChIP-seq
x		Flow cytometry
x		MRI-based neuroimaging

Human research participants

Policy information about studies involving human research participants

Population characteristics	Skeletal muscle biopsies were obtained from 53 very healthy GESTALT participants (22-83 yr, median=52 yr) who were defined as 'healthy' based on very strict inclusion criteria developed by the Clinical Research Unit of the National Institute on Aging. Our cohort was predominantly Caucasian (n=38) and male (n=33), and completed at least a high school education (n=30). In spite of the strict inclusion criteria, there were significant ($p<0.05$) differences in waist circumference (cm), fitness (VO2 max, ml/kg/min), fasting glucose (mg/dL), 400-m walking time (s), and knee strength (Nm) between younger (≤52 yr) and older (>52 yr) participants.
Recruitment	Subjects were recruited using multiple strategies, including but not limited to advertising in newspapers, flyers, Internet, radio, NIH and NIA websites, senior centers, senior expos and health fairs, volunteers who have expressed interest in NIA studies, and word of mouth from other study participants. All participants were recruited based on the same inclusion and exclusion criteria.
Ethics oversight	All participants underwent a consenting process that entailed a detailed description of the study, including potential risks. All participants signed an informed consent to participate in the study. The study protocol was approved by the National Institutes of Health (NIH) Institutional Review Board (IRB) and complied with all ethical regulations for work with human subjects.

Note that full information on the approval of the study protocol must also be provided in the manuscript.

Clinical data

Policy information about clinical studies

All manuscripts should comply with the ICMJEguidelines for publication of clinical research and a completedCONSORT checklist must be included with all submissions.

Clinical trial registration	NCT02339012
Study protocol	The full study protocol is provided at https://clinicaltrials.gov/ct2/show/study/NCT02339012?term=NCT02339012&rank=1.
Data collection	Muscle biopsies were collected from each participant in 2014, along with a number of physiological measures that typically change with aging, such as measures of body composition, measures of energy consumption and fitness, measures of energetic metabolism, measures of homeostatic equilibrium (hormones and inflammatory markers), measures of neuronal integrity and neuronal function, and measures of physical and cognitive function. The biopsies were collected at the NIA Intramural Clinical Research Unit in Baltimore, MD. RNA-sequencing was performed and analyzed at the Biomedical Research Center in Baltimore, MD.
Outcomes	This study aimed to elucidate differences in the transcriptomic network of skeletal muscle as a function of age using the state-of-the-art expression atlas from the GESTALT study. The primary outcome of this study is age and was self-reported at patient visits. There are no secondary outcomes in this study.
